# Reproducibility of three different cardiac T2-mapping sequences at 1.5T and impact of cofactors on T2-relaxation times

**DOI:** 10.1186/1532-429X-17-S1-W12

**Published:** 2015-02-03

**Authors:** Bettina Baessler, Frank Schaarschmidt, Bernhard Schnackenburg, Christian Stehning, Agathe D Giolda, David Maintz, Alexander Bunck

**Affiliations:** 1 Department of Radiology, University Hospital of Cologne, Cologne, Germany; 2 Institute of Biostatistics, Faculty of Natural Sciences, Leibniz Universität Hannover, Hannover, Germany; 3 Philips Healthcare Germany, Hamburg, Germany; 4 Philips Research Germany, Hamburg, Germany

## Background

The high interindividual variability of myocardial T2 relaxation times appears to be one of the main challenges for the clinical application of cardiac T2-mapping. This study therefore aimed to evaluate potential underlying causes for this variability, analyzing the reproducibility of three different cardiac T2-mapping sequences and evaluating the influence of cofactors on T2 relaxation times

## Methods

30 healthy volunteers were examined three times on a clinical 1.5T scanner (scan 1: in the morning; scan 2: in the evening of the same day; scan 3: in the evening 2-3 weeks later). In each examination three different T2-mapping sequences were acquired at three slices in short axis view: Multi Echo Spin Echo (MESE), T2-prepared balanced Steady State Free Precession (T2prep; [1]) and Gradient Spin Echo (GraSE). Repeated measurements were performed for T2prep and GraSE. Segmented T2-maps were generated for each slice according to the AHA 17-segment model. Intra- and inter-observer reproducibility was tested in a subgroup of 10 randomly selected subjects, where manual ROIs were drawn independently to measure T2 values of each segment blinded to the other results.

## Results

Overall, we observed no systematic difference of T2 times due to diurnal effects and on long-term analysis. Differentiated analysis of variance components for all sequences, however, revealed a greater variance of T2 times over multiple time points than for repeated measurements within the same scan. Our study revealed a low intra-observer and inter-observer variability of manual ROI-definition and the acquired T2 times for each sequence. The coefficients of variation and intraclass correlation coefficients for intra-observer variability were: 1.3% and 0.89 for T2prep, 1.5% and 0.93 for GraSE, 3.1% and 0.83 for MESE; and for inter-observer variability: 3.3% and 0.66 for T2prep, 2.0% and 0.83 for GraSE, 3.6% and 0.77 for MESE. With respect to the influence of potential cofactors on T2 times, we observed a negative effect of the cofactor heart rate on mean T2 values, yet this effect proved to be not significant. Conversely, we found significant and positive relation between mean T2 times and the cofactors age, weight and height (p < 0.005, p < 0.05 and p < 0.05) in single linear regression models. Using multiple regression models, we observed significant relations between mean T2 times and age (p < 0.005), gender (p < 0.01), and either weight or height (p < 0.005), for given values of the remaining cofactors.

## Conclusions

Intra- and inter-observer reproducibility of all tested T2-mapping sequnces is high, thereby confirming previous studies. According to our study, the high interindividual variability of myocardial T2 relaxation times is most likely due to proband-related effects such as age, gender, weight and height and other cofactors intraindividually varying with time.

## References

[B1] Giri S T2 quantification for improved detection of myocardial edema Journal of cardiovascular magnetic resonance 2009 11 56 10.1186/1532-429X-11-56 20042111 PMC2809052

